# Postoperative Complications in Women Submitted to a Transobturator Tape Sling: An 11-Year Retrospective Cohort

**DOI:** 10.7759/cureus.89924

**Published:** 2025-08-12

**Authors:** Henrique Rabelo Cortines, Rafael S Aguiar, Ricardo De Rizzo, Guilherme C Gonzales, Paula M Fonseca, Mariana C Graziano, Andre Coelho Pereira, Mateus Henrique Silva Faria, Vincius C Lopes, Ana Paula B Bogdan

**Affiliations:** 1 Department of Urology, Faculty of Medicine of São José do Rio Preto, São José do Rio Preto, BRA

**Keywords:** mid-urethral sling placement, sling, stress urinary incontinence, suburethral sling, transobturator tape, urinary incontinence

## Abstract

Introduction and hypothesis: Urinary incontinence (UI) is a common condition that impacts society. A transobturator tape (TOT) sling is one of the most widely used surgical options in the treatment of stress incontinence. This study aimed to investigate the incidence and types of postoperative complications, as well as the reoperation rate, in women undergoing TOT sling surgery.

Methods:A retrospective cohort study was conducted involving 194 women submitted to a TOT sling between 2010 and 2020 at São José do Rio Preto Base Hospital, a university hospital in the state of São Paulo, Brazil. Demographic characteristics, type of UI, preoperative urodynamic data, postoperative complications, and the need for reoperation were collected. The data were entered into a spreadsheet in MS Excel. IBM SPSS Statistics for Windows, version 23.0 (released 2014, IBM Corp., Armonk, NY) and GraphPad Instat (version 3.10, released 2009, GraphPad, California, USA) programs were used for the statistical analysis.

Results:The mean patient age was 56.4 years, and 68% of the sample had stress UI alone. The success rate was 77%, whereas 31% had clinical or surgical complications, such as de novo urgency and postoperative stress UI, mesh extrusion, urinary obstruction, and acute urinary retention. Only four patients (2%) required reoperation.

Discussion: The epidemiological findings (average age and prevalence of comorbidities, such as hypertension (36.6%) and diabetes (14.4%)) match the expected profile for women with UI. The overall complication rate was compatible with rates described in the literature, with a predominance of conservatively manageable clinical complications. Although 7.8% of the patients required intervention for surgical complications, the procedure proved safe and effective, even in a teaching environment.

Conclusions: TOT sling proved to be a safe, effective option for the treatment of stress UI, even in a training scenario. Most postoperative complications were managed clinically, and the reoperation rate was low. Despite the proven efficacy of the procedure, the monitoring of clinical complications is necessary to minimize the impact on the quality of life of patients.

## Introduction

Urinary incontinence (UI) is the involuntary loss of urine. This condition occurs throughout the world and has considerable social impacts. UI mainly affects women, with prevalence rates ranging from 5% to 70%, although most studies report rates between 25% and 45%. Among women 70 years of age or older, the prevalence of UI is 40% [1].

The different types are stress UI, which is characterized by loss associated with physical exertion, urge UI, characterized by loss associated with a sudden, intense desire to urinate, and mixed UI, which has characteristics of both previous types [2].

Treatment is generally gradual, beginning as less invasive and progressing to more invasive [3]. Affected individuals should initially be treated with lifestyle changes (weight loss, adequate water intake, programmed urination, cessation of smoking, etc.) combined with pelvic floor physiotherapy. In more complex or refractory cases, other therapies may be indicated, such as oral medication (antimuscarinic agents or β3 adrenergic receptor blockers), botulinum toxin, sacral neuromodulation, and even surgical procedures, such as midurethral sling, colposuspension, and urethral bulking agents [3,4].

Initially performed conservatively, treatment for stress UI can achieve improvements or even resolution in up to 70% of cases, but the adherence rate becomes progressively lower over time [5,6]. Thus, part of these patients will be submitted to a surgical procedure, such as a midurethral sling, which is currently considered the gold standard due to its efficacy and minimally invasive nature [7]. This procedure was developed and progressively improved over recent decades and is currently the most widely used for the correction of stress UI. The different types of midurethral slings include transobturator tape (TOT) and retropubic sling [8].

Despite the high success rate of slings, irrespective of the method, complications can occur, such as urinary infection, bleeding, chronic pain, mesh-related complications (e.g., infection or extrusion), trauma to the urethra or bladder, urinary obstruction, and de novo urgency [9]. 

The aim of the present study was to investigate the postoperative complication rate and need for reoperation in patients submitted to TOT sling surgeries performed between 2010 and 2020 at our service.

## Materials and methods

A retrospective cohort study was conducted involving women with stress UI submitted to TOT midurethral sling surgery with synthetic material performed by the team of the Urology Service of the São José do Rio Preto Base Hospital in the state of São Paulo, Brazil, through the Brazilian public healthcare system in the period from 2010 to 2020. This study received approval from the institutional review board (certificate number: 82831824.9.0000.5415).

All patients with stress UI refractory to conservative treatment or who did not respond to it were recommended a transobturator sling surgery. Those patients who underwent the procedure during the study period were included, except those who met some exclusion criteria. Having previously been submitted to midurethral sling at a different service, cases of retropubic and autologous retropubic sling, vaginal prolapse, and neurogenic disease were the exclusion criteria. Data were collected from a meticulous review of the electronic charts available in the MVPep computation system of the São José do Rio Preto Regional School of Medicine Foundation (FUNFARME). 

After excluding the patients who did not meet all the above criteria, the number of patients analyzed was 194. We collected data from the electronic chart of those patients. We read all the previous appointments and extracted data including age, comorbidities (diabetes mellitus, arterial hypertension, psychiatric disease (depression, anxiety), heart disease, fibromyalgia, hypothyroidism, previous physiotherapy (yes or no), presence of urgency before and after surgery, Valsalva leak point pressure (VLPP) in cmH2O, de novo stress and urgent UI, preoperative and postoperative detrusor overactivity, infravesical obstruction, acute urinary retention, urinary tract infection, mesh extrusion and erosion, whether reoperation occurred and type of sling during reoperation, and success rate (absence of UI after sling proven through clinical examination).

The data were entered into a spreadsheet in Microsoft Excel (Microsoft® Corp., Redmond, WA). Descriptive statistical analysis was performed, with the calculation of frequencies, as well as central tendency and dispersion measures. IBM SPSS Statistics for Windows, version 23.0 (released 2014, IBM Corp., Armonk, NY) and GraphPad Instat (version 3.10, released 2009, GraphPad, California, USA) programs were used for the statistical analysis.

## Results

The sample was composed of 194 patients submitted to a midurethral sling (Table 1). The mean age was 56.4 years (range: 32-82 years). Urodynamic studies were performed on all patients prior to the procedure, following the institutional protocol. The mean Valsalva leak point pressure (VLPP) was 73.6 cmH20 (range: 10-257 cmH_2_0).

**Table 1:** Descriptive statistics for age and VLPP VLPP: Valsalva leak point pressure

Metric		Age (years)		VLPP (cmH₂O)
Mean		56.42		73.26
Standard deviation		9.90		44.77
Median		56.00		62.50
Minimum		32.00		10.00
Maximum		82.00		257.00

A total of 132 patients (68%) had stress UI, and 62 (32%) had mixed (Table 2). Most patients (52%, n = 101) had undergone physiotherapy prior to the procedure. Comorbidities were found in 103 patients (53%) (Table 3). The most prevalent was hypertension (36.6%, n = 71), followed by diabetes mellitus (14.4%, n = 29). The prevalence of psychiatric diseases among the patients studied was low (6.7%, n = 13).

**Table 2:** Presurgical diagnosis x frequency UI: urinary incontinence

Diagnosis	Frequency (n)	Percentage (%)
Stress UI	132	68.0
Mixed UI	62	32.0
Total	194	100.0

**Table 3:** Percentage distribution of comorbidities in patients submitted to a transobturator tape (TOT) sling (n = 194). N: number of individuals; * depression, anxiety

Comorbidity	N	%
Hypertension		
Absent	123	63.4
Present	71	36.6
Diabetes mellitus		
Absent	165	85.1
Present	29	14.9
Psychiatric disease *		
Absent	181	93.3
Present	13	6.7
Heart disease		
Absent	184	94.8
Present	10	5.2
Hypothyroidism		
Absent	179	92.3
Present	15	7.7
Fibromyalgia		
Absent	180	92.8
Present	14	7.2

The success rate, defined as the absence of any type of UI after sling procedure, proven by the clinical evaluation and patient report, was 77% (n = 150). The prevalence of postoperative stress and urgent UI was 16% (n = 31) and 13.4% (n = 26), respectively (Table 4).

**Table 4:** Percentage distribution of urodynamic data and symptoms in patients submitted to TOT sling (n = 194). UI: urinary incontinence, TOT: transobturator tape *This item had not been described in the urodynamic report.

Condition	N	%
Detrusor overactivity		
Absent	147	75.8
Present	28	14.4
Not informed*	19	9.8
De novo urgency		
Absent	163	84.0
Present	31	16.0
Postoperative urgent UI		
Absent	168	86.6
Present	26	13.4
Postoperative stress UI		
Absent	163	84.0
Present	31	16.0
De novo detrusor overactivity		
Absent	106	54.6
Present	2	1.0
Not informed*	86	44.3

Complications were classified as clinical (Table 5) and surgical (Table 6). Clinical complications were resolved exclusively through conservative approaches, with no need for invasive interventions. Surgical complications encompass those requiring some type of procedure, either minimally invasive or surgical. The most frequent clinical complications were de novo urgency and postoperative stress UI, affecting 16% (n = 31) of patients, followed by postoperative urgent UI (13.4%, n = 26). The most prevalent surgical complications were mesh extrusion (4.6%, n = 9), urinary obstruction (2.6%, n = 5), the need for reoperation (2.6%, n = 4), acute urinary retention (0.5%, n = 1), and mesh erosion (0.5%, n = 1). The total complication rate (clinical and surgical) was 31% (n = 60) (Table 7). Only four patients (2%) were submitted to reoperation. The reoperation procedures were TOT (n = 2), autologous retropubic sling (n = 1), and synthetic retropubic sling (n = 1).

**Table 5:** Percentage distribution of clinical complications after TOT sling (n = 194) UI: urinary incontinence, TOT: transobturator tape

Complication	N	%
De novo urgency		
No	163	84.0
Yes	31	16.0
Postoperative urgent UI		
No	168	86.6
Yes	26	13.4
Postoperative stress UI		
No	163	84.0
Yes	31	16.0
De novo detrusor overactivity		
Not informed	86	44.3
No	106	54.6
Yes	2	1.0
Repeated urinary tract infection		
Absent	187	96.4
Present	7	3.6

**Table 6:** Percentage distribution of surgical complications after TOT sling (n = 194). TOT: transobturator tape

Complication	N	%
Postoperative obstruction		
Absent	189	97.4
Present	5	2.6
Acute urinary retention		
Absent	193	99.5
Present	1	0.5
Mesh extrusion		
Absent	185	95.4
Present	9	4.6
Mesh erosion		
Absent	193	99.5
Present	1	0.5
Reoperation		
Absent	190	98
Present	4	2
Type of reoperation		
Not performed	190	97.9
Autologous retropubic sling	1	0.5
Synthetic retropubic sling	1	0.5
TOT	2	1.0

**Table 7:** Percentage distribution of patients with post-TOT sling complications (n = 194). TOT: transobturator tape

Complications	N	%
Total complications		
Absent	134	69
Present	60	31
Clinical complications		
Absent	141	72.6
Present	53	27.4
Surgical complications		
Absent	179	92.2
Present	15	7.8

## Discussion

A midurethral sling is widely available at urology services throughout the world and is currently considered the gold standard for the treatment of stress UI, with a progressive increase in the number of surgeries since the 1990s due to the excellent outcome and low surgical morbidity rate. In a meta-analysis, Chen et al. [10] found a success rate of 73.8% among patients submitted to a transobturator sling. Karmakar et al. [11] and Sabadell et al. [12] reported success rates of 71.6% and 77.6%, respectively, which is similar to the rate found in the present investigation (77%). Treatment success in this study was defined as the absence of postoperative UI. 

According to the institutional protocol at our service, all patients to be submitted to any surgery for UI should undergo a prior urodynamic exam. Despite not being mandatory or significantly altering the postoperative outcome [13], this exam is considered essential by many urologists, as it assists in the diagnosis of factors, such as asymptomatic detrusor overactivity or urinary disorder, which can assist in predicting postoperative outcomes [14]. In more complex cases, urodynamics can lead to the modification or even the cancellation of surgery [15].

With regard to VLPP, this urodynamic variable is not directly related to the success of conservative treatment or even a precise indication for surgery. However, in some studies found in the literature, such as that conducted by Jung et al. [16], preoperative VLPP ≥ 75 cmH_2_O has 95.7% sensitivity and a positive predictive value of 98.4% in predicting the success of surgery. 

However, it is important to highlight that urodynamic studies, despite their utility, have significant limitations for patients with stress UI. The test is invasive, uncomfortable, and expensive. Furthermore, the testing environment does not replicate real-life conditions and may not accurately reflect a patient's true symptoms [17].

With regard to epidemiological factors, the average patient age was similar to that found in the literature for patients submitted to the sling procedure. According to an American study involving 36,195 women submitted to midurethral sling, the average age was 53.7 years [18], placing patients in the sixth decade of life, as occurred in the present investigation (average of 56.4 years). The rate of comorbidities in the present sample was also expected, such as 36.6% for hypertension and 14.4% for diabetes mellitus. [19]

Psychiatric diseases, such as depression and anxiety, are reported to be more prevalent among women with UI [20]. In the present study, the prevalence of these conditions was only 6.7%. By contrast, Steibliene et al. [21] analyzed 60 women with stress UI and found a significantly higher rate, as 30% of patients had some degree of psychiatric impairment. With regard to fibromyalgia, although some authors report a greater frequency of pelvic disorders and symptoms of the lower urinary tract, there is no evidence of a direct association between this disease and stress UI [22].

The literature reports complication rates ranging from 10.5% to 31.3% [23], which is very close to the rate found in the present study (31%). The rate of mesh extrusion and/or exposure was 4.6%, which is similar to the 2.4% reported by Brazzelli et al. [24]. De novo urgency is one of the factors that can exert an impact on the quality of life of these patients. Symptoms of urgency are directly related to the perception of a cure in patients following the sling procedure. In the present study, the rate of de novo urgency was 16%. Lo et al. [25] investigated the occurrence of de novo detrusor overactivity and symptoms of urgency in women treated with midurethral sling, reporting rates of de novo urgency ranging from 11% to 25% higher. Some factors, such as increased sensitivity, low vesical capacity, age 66 years or older, diabetes mellitus, and greater urinary loss, may be associated with this result [25].

A limitation of the study is the failure to assess postoperative pain, including dyspareunia or chronic pelvic pain. Pain is described as one of the most common complications after TOT sling, although it is rare after six weeks [26]. Since the study was conducted with a review of medical records, this information was limited in most patients. Likewise, another limitation is to specify psychiatric conditions as the patient mostly does not know exactly the disease they have been diagnosed.

## Conclusions

The present study involved nearly 200 cases over an 11-year period. Although complications occurred in 31% of cases, most were managed clinically, with no need for invasive procedures. Only 7.8% required surgical interventions, and the sling reoperation rate was low (2%). Despite the low frequency of surgical complications and reoperation, 27% of the patients had clinical complications, which, although generally manageable without invasive interventions, can exert a negative impact on the lives of patients, underscoring the need for careful follow-up, even in a teaching scenario with high standardization.

A midurethral sling is widely recognized as the gold standard for the treatment of stress UI. Despite its efficacy, the procedure is not free of complications, which may be influenced by the patient’s age and comorbidities.
